# Mid Infrared Optical Gas Sensor Using Plasmonic Mach-Zehnder Interferometer

**DOI:** 10.1038/s41598-020-57538-1

**Published:** 2020-01-28

**Authors:** Raghi S. El Shamy, Diaa Khalil, Mohamed A. Swillam

**Affiliations:** 1 Department of Physics, School of Science and Engineering, The American University in Cairo, New Cairo, 11835 Egypt; 2 Department of Electronics and Communication, Faculty of Engineesring, Ain Shams University, Abassia, Cairo, 11517 Egypt

**Keywords:** Optics and photonics, Applied optics, Optical materials and structures

## Abstract

In this work, we propose an optimized design for on-chip gas sensor using metal-insulator (MI) plasmonic waveguide in the mid infrared range and utilizing a Mach-Zehnder Inetrferometer (MZI). The MI waveguide utilizes a high index dielectric layer on top of the metal to enhance the sensitivity of the sensor. The thickness and the refractive index of this layer are optimized to achieve high sensitivity. Using this layer, a design that exhibits high performance for both wavelength and intensity interrogation schemes is achieved. In addition, another one that furtherly enhances the sensor performance for intensity interrogation is also proposed. This design also minimizes the sensor sensitivity to wavelength variations. Intensity interrogation scheme has the advantage of eliminating the size and cost needed by wide wavelength band measurements including either spectrometer or tunable laser in wavelength interrogation. The first design sensitivity has reached 10000 nm/RIU with wavelength interrogation figure of merit (FOM_λ_) of 133RIU^−1^ and intensity interrogation FOM_I_ of 239RIU^−1^. While the second one exhibit FOM_I_ of 363RIU^−1^, both with length of 250 µm around 4.6 µm wavelength. Finally, these structures are cheap, compact, and easy to fabricate.

## Introduction

Mid infrared region is recently attracting a great attention as due to the wide range applications. These applications include; thermal imaging, infrared spectroscopy, chemical and biological sensing^1–3^. The importance of this spectral region arises from the fact that many chemical and biological molecules have their characteristic absorption within this region^4^. Optical detection of molecules is based on intensity or wavelength change due to change in the real or imaginary part of the analyte refractive index. Both imaginary and real parts of the refractive index, of the molecules under detection, exhibit a peak around its characteristic absorption wavelength. Hence, amplifying the effect that is detected by the optical device. This fact makes mid-infrared a suitable range for biomolecular and gas detection with high sensitivity. Gases like methane CH_4_, carbon dioxide CO_2_, and carbon monoxide CO have high absorption around 3.2 µm, 4.3 µm and 4.6 µm wavelengths, respectively.

Many refractive index gas sensors – refractometers – were proposed recently^5–8^, where the real part (n) of the refractive index is detected. Refractive index sensors have the advantage of ultra-small sample volume as its sensitivity does not depend on the sample volume, and hence are promising for integrated on chip sensors^9^. Most of the proposed refractive index gas sensors are working in the near infrared, and hence do not benefit from the signal amplification that happens to the gas refractive index in the MIR.

In general, there are two main sensing schemes for the refractive index sensors; the wavelength interrogation, and the intensity interrogation^10^. The wavelength interrogation scheme is based on the resonant wavelength shift with the change in the analyte refractive index. On the other hand, the intensity interrogation scheme is based on the intensity shift at a certain detection wavelength. Detecting the real part of the refractive index using wavelength interrogation requires either a spectrometer or a tunable laser, which will add to the size and cost of the sensor. However, many of the previously proposed refractive index gas sensors utilized wavelength interrogation^5–8^.

Surface plasmon resonance SPR sensors have achieved very high sensitivity, up to 13800 nm/RIU^11^, using the well-known method of attenuated total reflection (ATR) proposed by *Kretschmann*^12,13^. However, the large sizes of the SPR sensors prohibit them from being integrated on a single chip, as usually required for producing portable low-cost sensors. Moreover, the SPR sensors require careful alignment, thus, they lack strongly needed advantages such as rapid and high throughput measurements. Many efforts have been done to overcome these drawbacks, using nanoplasmonic structures like nanoparticles, nanoslit arrays and nanohole arrays^14–16^, to obtain low cost, small size and high throughput sensors. However, these efforts are lacking the high sensitivity that can be achieved using the conventional SPR sensor, with best sensitivity of 560 nm/RIU obtained by the nanoslits.

Mach-Zehnder Interferometer (MZI) is one of the well-known label free optical sensing devices, which can achieve high sensitivity^17–19^. Plasmonic waveguides with different configurations such as Metal-Insulator (MI)^12^, Insulator-Metal-Insulator IMI^20,21^ and Metal-Insulator-Metal (MIM)^22–25^, as well as plasmonic directional couplers^26^ can be used to form plasmonic MZI. Plasmonic MZI sensors were recently proposed for ultrasensitive on chip biosensing, with sensitivity reaching 3695 nm/RIU at 730 nm wavelength, and device length of 57.6 µm^27–29^. Beside the high sensitivity, this design forms on-chip easy-to-fabricate sensor. This sensor is capable for rapid, portable and high throughput operation using multiplexed array sensing. Finally, it can be integrated with microfluidic channel on the same chip.

Many difficulties are faced when trying to design plasmonic gas sensors in the MIR range. Plasmonic waveguide sensitivity is proportional to refractive index to be sensed, and inversely proportional to the operating wavelength. In this paper a MIR gas sensor is proposed using the vertical MZI structure in^29^. High sensitivity up to 10000 nm/RIU and high figure of merit (FOM) are achieved. To achieve this performance, a high index layer is used above the metal of the sensing arm. The sensor analysis and design are done using finite difference time domain (FDTD) solver^30^. Two sensor designs are proposed; the first design is optimized for wavelength interrogation like many of the previously published refractive index sensors^5–8,27–29^, while the second design is optimized for intensity interrogation scheme, which possesses the advantages of high compactness and low cost. These designs have all the advantages of the previously proposed liquid MZI sensor^29^, in addition to, its high sensitivity to gaseous medium and operation in the mid infrared, around the characteristic absorption of gases.

In section II, the plasmonic MZI sensor structure is presented together with the sensor performance parameters and the design approach. Section III proposes the metal-insulator (MI) waveguide with high index layer to enhance the performance of the plasmonic MZI sensor. Section IV is devoted for the implementation of the gas sensor designs, and the finite difference time domain (FDTD) simulation results and optimization. Finally, the conclusion is given in section V.

## Results and Discussions

### Structure and MZI analysis

**Figure 1:**
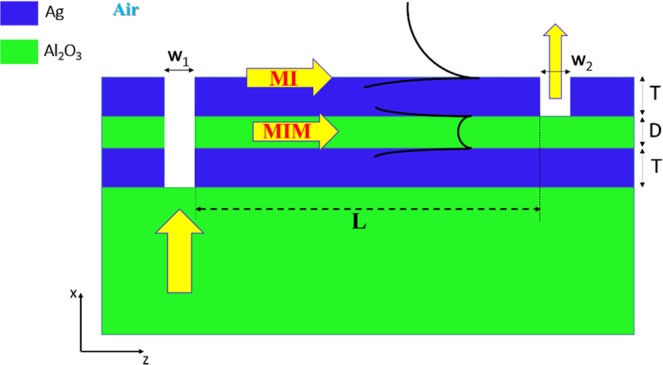
Vertical Plasmonic MZI with MIM and MI arms.

The proposed structure consists of three layers: metal-sapphire-metal above a sapphire substrate, which forms MIM and MI waveguides that construct the MZI reference and sensing arms, respectively, see Fig. 1. The sapphire is chosen due to its low absorption in the wavelength range 1.1–6 µm^31^, and the metal used is silver (Ag). The input plane wave, from the substrate, is coupled to the MIM and MI waveguide modes through the input slot of width w_1_, then each mode propagates with its propagation constant (β) distance L, and finally coupled out through the output slot w_2_ and interfere with each other.

The output signal intensity is given by:

1‐a $$\frac{{I}_{out}}{{I}_{in}}={(A+B)}^{2}-4AB\,{\sin }^{2}(\frac{2\pi ({n}_{effMI}-{n}_{effMIM})}{\lambda }\frac{L}{2})$$

With resonant wavelength λ_res_ and FSR_λ_ equal to:

1‐b $${{\rm{\lambda }}}_{{\rm{res}}}=({\Delta n}_{{\rm{eff}}}\ast {\rm{L}})/q,$$

1‐c $${{\rm{FSR}}}_{{\rm{\lambda }}}={{\rm{\lambda }}}^{2}/({\Delta n}_{{\rm{eff}}}\ast {\rm{L}})$$

and

1‐d $$\Delta {n}_{eff}={n}_{eff-MI}-{n}_{eff-MIM}$$

where q is an integer number, Δn_eff_ is the difference between the MI and MIM effective indices, A = a_1_a_2_exp(−γ_1_L) and B = b_1_b_2_exp(−γ_2_L) are the normalized output power of MIM and MI mode respectively, with a_1_,a_2_ and b_1_,b_2_ and γ_1_, γ_2_ are the input, output coupling coefficients and losses of MIM and MI modes, respectively.

One can easily derive the MZI sensitivity (dλ/dn) and the full width half maximum (FWHM) from (1) and get:

2 $$S=\frac{d\lambda }{dn}=\frac{\lambda }{\Delta {n}_{eff}}\frac{{d}_{neffMI}}{dn}=\frac{\lambda }{\Delta {n}_{eff}}{S}_{wg}$$

where S_wg_ = dn_eff-MI_/dn is the MI waveguide sensitivity.

3 $$FWH{M}_{\lambda }=\frac{{\lambda }^{2}}{\pi L({n}_{effMI}-{n}_{effMIM}\,)}\frac{A+B}{\sqrt{2AB}}=\frac{FS{R}_{\lambda }}{\pi }\frac{A+B}{\sqrt{2AB}}$$

It can be deduced from (2) that the MZI sensitivity increases linearly by increasing the wavelength; however, the FWHM increase with wavelength is quadratic. In the wavelength interrogation method, the minimum detected refractive index change Δn is determined by the FWHM. While, in intensity interrogation method, the sensor performance is determined mainly by intensity changes. Thus, for better performance sensor, a figure of merit FOM is defined. For wavelength interrogation scheme, the FOM is defined as^28^:

4 $$FO{M}_{\lambda }=\frac{d\lambda /dn}{FWH{M}_{\lambda }}=\frac{\pi L}{\lambda }{S}_{wg}\frac{\sqrt{2AB}}{A+B}$$

And for intensity interrogation scheme, the FOM is defined as^29^:

5‐a $$FO{M}_{I}=\frac{1}{I}\frac{dI}{dn}$$

From (1) we get:

5‐b $$\frac{dI}{dn}\propto 2\,\sin (\frac{\Delta \phi }{2})\cos (\frac{\Delta \phi }{2})\frac{d\Delta \phi }{dn}=\,\sin (\Delta \phi )\frac{\pi L}{\lambda }{S}_{wg}$$

For A = B we will get:

5‐c $$FO{M}_{I}\propto \,\sin (\Delta \phi )\frac{d\lambda /dn}{FWH{M}_{\lambda }}=\,\sin (\Delta \phi )FO{M}_{\lambda }$$

Equation (5-c) shows that optimizing FOMI is also optimizing FOM_λ_. Hence, to achieve high detection performance we have to: increase the MI waveguide sensitivity S_wg_, decrease waveguides losses, equalize both modes output power (A = B), for minimum FWHM, increase MZI length, and work at low wavelengths. To suppress the effect of different wavelength regions when comparing with other designs, we will also compare the term L/λ. So, in our design, we are trying to increase the sensitivity of the MI waveguide, decrease waveguides losses and balance the modes output power.

### Reference arm: Metal-Insulator-Metal waveguide performance

**Figure 2:**
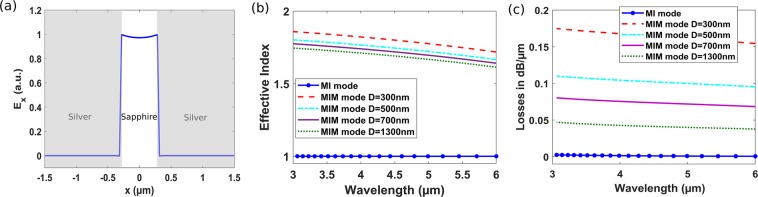
(**a**) MIM waveguide mode major component E_x_ with silver, sapphire and D = 600 nm. (**b**) Effective index and (**c**) Losses of the MIM waveguide versus wavelength at different slot widths D together with the MI waveguide of gaseous medium.

In principle, MIM waveguide support two TM modes one with symmetric and another one with antisymmetric transversal electric field component (E_x_)^12,22,23^. However, the symmetric mode is of more interest as the antisymmetric mode suffers from low confinement and exhibit cut-off at small slot widths^12^. As mentioned previously, MIM waveguide is the reference arm of our MZI sensor, with silver and sapphire as the metal and insulator, respectively. The symmetric mode profile of our MIM waveguide with D = 600 nm is shown in Fig. 2a. Our MIM waveguide exhibit higher effective index compared to the MI sensing arm (which is almost one for gaseous medium) due to the high field confinement within the slot^24,25^, see Fig. 2b. MIM mode effective index increases as the slot width and wavelength decrease, Fig. 2b. In general, operating at wavelengths closer to the metal plasma wavelength increase the confinement and hence the effective index of the plasmonic waveguides^12^; the silver used here has plasma wavelength around 280 nm^32^. However, as effective index increases as the MIM mode intrinsic losses increase significantly compared to MI mode losses (which is around 0.001 dB/µm for gaseous medium), see Fig. 2c.

### Sensing arm: Metal-Insulator waveguide performance

The design of high performance plasmonic MZI gas sensor in the MIR region using MI waveguide as the sensing arm has two main challenges: 1) The MI waveguide sensitivity (S_wg_) is small for two reasons. Firstly, the sensitivity decreases as the insulator index decreases. So, for gas sensing, the sensitivity of the MI waveguide is lower than that of the higher index biomolecules; liquid sensing. Another reason is that the sensitivity of the waveguide decreases as the operating wavelength gets far from the metal plasma resonance wavelength. 2) MI waveguide with low-index gas as the insulator material results in MI waveguide losses much lower than that of the MIM waveguide, see Fig. 2c, which according to (3) increases the FWHM (as A ≪ B, while minimum at A = B). Hence, according to (4) this will result in low FOM as the two main parameters S_wg_ and FWHM are deteriorated.

**Figure 3:**
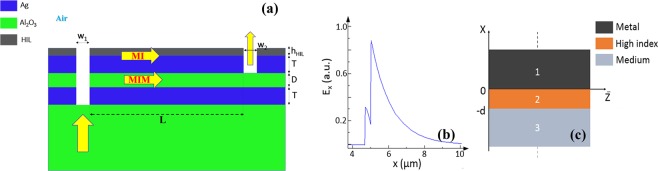
(**a**) Vertical plasmonic MZI with high index layer (HIL), (**b**) MII waveguide mode major component E_x_ with silver and HIL with index 2.4 and thickness 320 nm at λ = 4.5 µm with metal/HIL interface at x = 4.7 µm, (**c**) MII waveguide.

To overcome all these issues at once, a high index layer (HIL) is introduced above the metal of the MI waveguide, as shown in Fig. 3a, forming MII waveguide^33–35^. Using Maxwell equations, we can obtain the dispersion relation of the TM mode in a metal-insulator-insulator (MII) waveguide, Fig. 3b,c, as follows^36^:

6‐a $${k}_{0}d=\frac{{\tan }^{-1}(f)+{\tan }^{-1}(g)}{{u}_{2}}$$

with

6‐b $$f=\frac{{\varepsilon }_{2}{u}_{1}}{{\varepsilon }_{1}{u}_{2}}$$

and

6‐c $$g=\frac{{\varepsilon }_{2}{u}_{3}}{{\varepsilon }_{3}{u}_{2}}$$

and

6‐d $${u}_{1}=\sqrt{{n}_{eff}^{2}-{\varepsilon }_{1}},\,{u}_{2}=\sqrt{{\varepsilon }_{2}-{n}_{eff}^{2}},\,{u}_{3}=\sqrt{{n}_{eff}^{2}-{\varepsilon }_{3}}$$

where d is the HIL thickness, ε_1_ = ε_m_ is the metal permittivity, ε_3_ ≈ 1 the sensing medium permittivity and ε_2_ > ε_3_ is the high index layer permittivity, n_eff_ the waveguide effective index, k_0_ the free space wavenumber.

**Figure 4:**
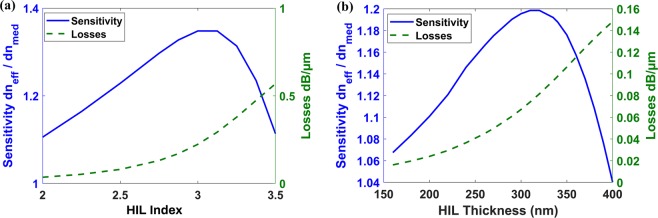
Sensitivity and Losses of the MI waveguide versus: (**a**) HIL refractive index with h_HIL_ = 250 nm at λ = 4 µm and (**b**) Si_3_N_4_ thickness (h_HIL_) at λ = 4.5 µm.

Using the previous analytical expression, we calculate the MII waveguide sensitivity for different HIL thicknesses and refractive indices. The analysis shows that this layer should be optimized regarding its thickness and index at the operating wavelength, because for large thickness and/or index of the layer, the MI mode field is confined mostly inside this layer, and hence the sensitivity decreases due to the weak field in the sensing medium. So, there is an optimum thickness and index for this layer to enhance the sensor performance. Therefore, we firstly perform modal analysis using finite difference solver to optimize the thickness and index of this layer for maximum sensitivity. Initial optimization shows that for 250 nm thickness of this HIL, the highest sensitivity is achieved at index of 3 for λ = 4 µm, as depicted in Fig. 4a. We, therefore, use silicon nitride Si3N4^37^ for this layer with refractive index of 2.4 around 4.5 µm wavelength. When optimizing for the highest sensitivity at 4.5 µm wavelength, near the absorption peak of CO and CO2, the thickness of Si_3_N_4_ is 320 nm, Fig. 4b.

**Figure 5:**
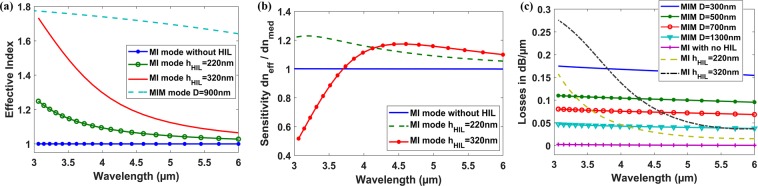
(**a**) Effective index, (**b**) Sensitivity versus wavelength of MI waveguide at different HIL thickness and MIM waveguide with D = 900 nm. (**c**) Losses of the MIM waveguide at different insulator thickness D and MI waveguide at different HIL thickness versus wavelength.

The analysis shows that this HIL will solve the two main challenges mentioned previously. This layer will: 1) increase the effective index and more importantly the sensitivity (S_wg_) of the MI waveguide, as shown in Fig. 5a,b, respectively. 2) increase the losses of the MI waveguide and makes it comparable to that of the MIM (A ≈ B), see Fig. 5c, such that the FWHM according to (3) is minimized. Consequently, this will enhance the FOM significantly according to (4). Note that wavelength with higher sensitivity suffers also from higher losses. Hence, the optimum wavelength of operation, with highest FOM not sensitivity, is achieved through FDTD optimization.

### Wavelength interrogation design

From Fig. 4b, we get that for maximum sensitivity, the thickness of the Si_3_N_4_ must be h = 320 nm at λ = 4.5 µm, see Fig. 4b. Now we want to design the MZI using this MII waveguide. For λ = 4.5 µm, we choose D = 700 nm, w_1_ = 2400 nm and w_2_ = 1000 nm in order to have efficient coupling and also equal power in both modes. For the two waveguides modes to be uncoupled, the thickness of the metal T has to be greater than 1400 nm at λ = 4.5 µm. We initially choose T = 1800 nm and the MZI length to be L = 100 µm.

**Figure 6:**
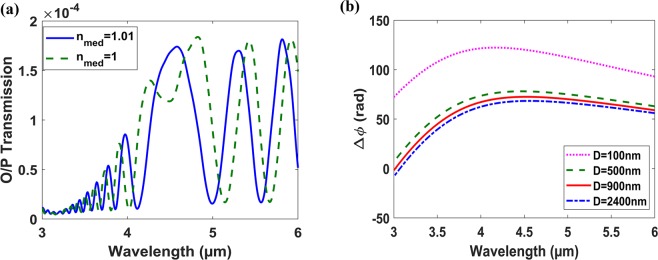
(**a**) Normalized Output transmission versus wavelength of the MZI with D = 700, T = 1800 nm, h_HIL_ = 320 nm, w_1_ = 2400 nm, w_2_ = 1000 nm and L = 100 µm. (**b**) Phase difference Δφ at different MIM thickness (D) with h_HIL_ = 320 nm versus wavelength.

Figure 6a shows the FDTD results of the designed MZI. The sensor performance is degraded, exhibits large FWHM, around the wavelength of maximum sensitivity, λ = 4.5 µm. This is due to the phase difference, Δφ = (2π/λ)*Δn_eff_*L, of the MZI waveguides. This phase difference saturates and exhibits a maximum around 4.5 µm wavelength, as shown in Fig. 6b. Also, the maximum transmission does not occur due to Δφ_res_ = 2qπ, i.e. the resonant wavelength condition, where q is integer. However, it always happens at the wavelength of maximum Δφ. Thus, resulting in almost zero wavelength shift (dλ/dn ≈ 0) and accordingly very low FOM_λ_, (4).

We can change this response, along the wavelength, by changing waveguides dimensions. Further increase in the MIM waveguide thickness D does not result in shifting this behavior to wavelengths far from the maximum sensitivity wavelength. However, decreasing D to 100 nm or lower results in shifting Δφ maximum wavelength to lower values, but this very small thickness D increases the MIM waveguide loss significantly as well, around 0.5 dB/µm for D = 100 nm, resulting in very weak resonance. To solve this issue, we can work on higher operating wavelength, or change the HIL thickness (h_HIL_) and sacrifice the maximum sensitivity, see Figs. 4b, 5b. However, changing the HIL layer thickness to lower values is better as it results in decreasing the MI waveguide loss.

**Figure 7:**
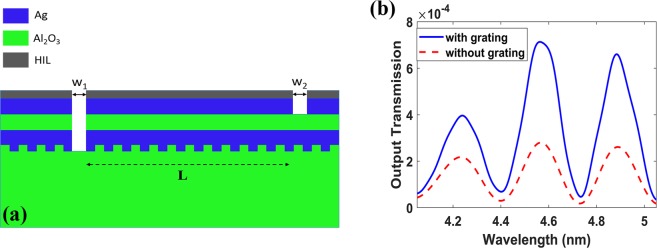
(**a**) Vertical plasmonic MZI with high index layer and grating. (**b**) Normalized Output transmission versus wavelength with grating P_gr_ = 1216.2 nm, h_gr_ = 475 nm and without grating with D = 900 nm, T = 1500 nm, h_HIL_ = 240 nm, w_1_ = 1550 nm, w_2_ = 1600 nm and L = 250 µm.

Moreover, the output transmission is low and need to be increased. Thus, we used a grating on the substrate-metal interface, as shown in Fig. 7a to increase the input power coupling and hence the output power. The optimized grating dimensions are P_gr_ = 1216.2 nm, the grating period and h_gr_ = 475 nm, the grating thickness, see Fig. 7b. We then re-optimized the input and output slots, and get w_1_ = 1550 nm and w_2_ = 1600 nm. This enhanced the output power of the initial design, Fig. 6a, by a factor of 3.6. Then, further optimization is done using FDTD simulations to maximize the FOM and select the suitable operating wavelength. Note that, for the MIM to support single mode, the insulator layer thickness (D) must be lower than 1400 nm at 4.5 µm wavelength.

**Figure 8:**
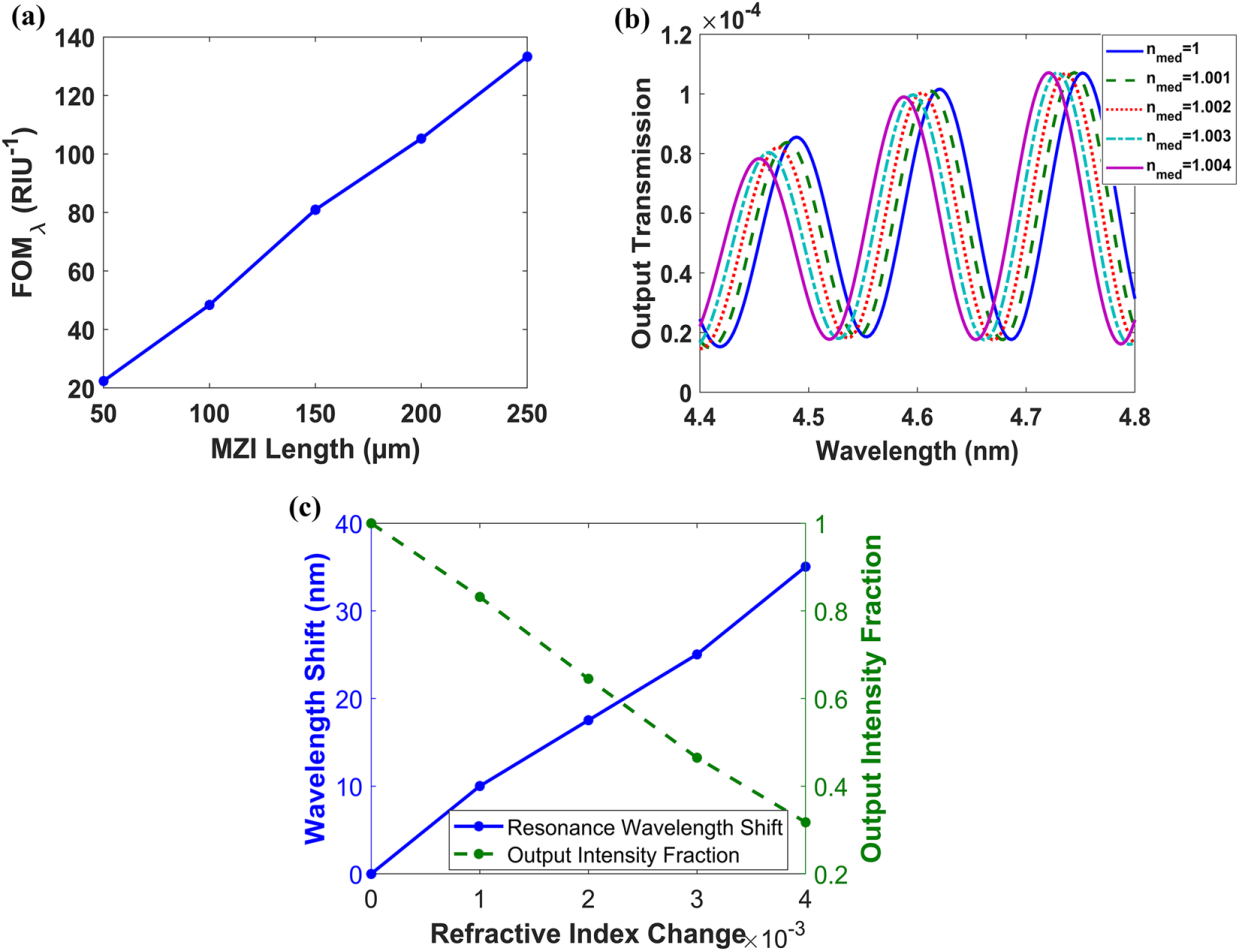
Vertical plasmonic MZI with D = 900 nm, T = 1500 nm, h_HIL_ = 240 nm, w_1_ = 1550 nm, w_2_ = 1600 nm, P_gr_ = 1216.2 nm and h_gr_ = 475 nm. (**a**) Figure of Merit (FOM_λ_) versus MZI length around λ = 4.6 µm. (**b**) Normalized output transmission versus wavelength at different medium refractive indices at L = 250 µm. (**c**) Resonance wavelength shift and output intensity fraction versus air medium refractive index change with MZI length of 250 µm around λ = 4.6 µm.

After FDTD optimization, the design with D = 900 nm, T = 1500 nm and h_HIL_ = 240 nm at L = 100 µm reaches FOM_λ_ of 48.4RIU^−1^, around 4.6 µm wavelength. The length of the MZI is fixed at 100 µm, which is only 22 times the operating wavelength. FOM increases as the MZI length increases (4) and (5). As mentioned previously, when comparing between different MZI designs, it is important to compare designs with the same L/λ ratio. The previously published results for similar MZI^28^, working as a liquid sensor, was FOM_λ_ of 122RIU^−1^ and 150RIU^−1^ with L/λ ratio of 50 and 63.2, respectively, and wavelength around 700 nm. Figure 8a shows the FOM_λ_ versus MZI length for our design, with maximum length of 250 nm which corresponds to L/λ = 54. At L = 250 µm, Fig. 8b, our sensor reaches FOM_λ_ of 133RIU^−1^, around 4.6 µm wavelength, with S = 10000 nm/RIU, FWHM = 75 nm and FOM_I_ of 239 RIU^−1^ for refractive index change Δn = 1e-3. Figure 8c shows the resonance wavelength shift and the output intensity change at different air medium refractive index change, with MZI length of 250 µm.

### Intensity interrogation design

Further investigation shows that the region where the Δφ saturates and exhibits a maximum, see Fig. 6, can result in high FOM_I_ by selecting the appropriate MZI length. Although the FWHM is large and the wavelength shift Δλ is almost zero, which result in very bad FOM_λ_. This is because FOM_I_ depends only on the waveguide sensitivity (S_wg_) of the sensing arm and the MZI length (5-b). As mentioned above, the maximum transmission always happens at the wavelength of maximum Δφ. Hence, the intensity change will not happen due to wavelength shift in the resonant condition, but due to the value of the Δφ at the same wavelength. Also, working at this wavelength where the FWHM is increased, has another advantage; at this wavelength region the intensity change is less dependent on the output wavelength of the source. Hence, optimization is done using FDTD simulations for the MI waveguide that exhibit maximum waveguide sensitivity around 4.5 µm with h_HIL_ = 320 nm. The optimized design dimensions are: D = 1000 nm, T = 2000 nm, h_HIL_ = 320 nm, w_1_ = 2100 nm and w_2_ = 1800 nm. Again grating with P_gr_ = 918.4 nm and h_gr_ = 450 nm is used to enhance the output power around 4 times.

**Figure 9:**
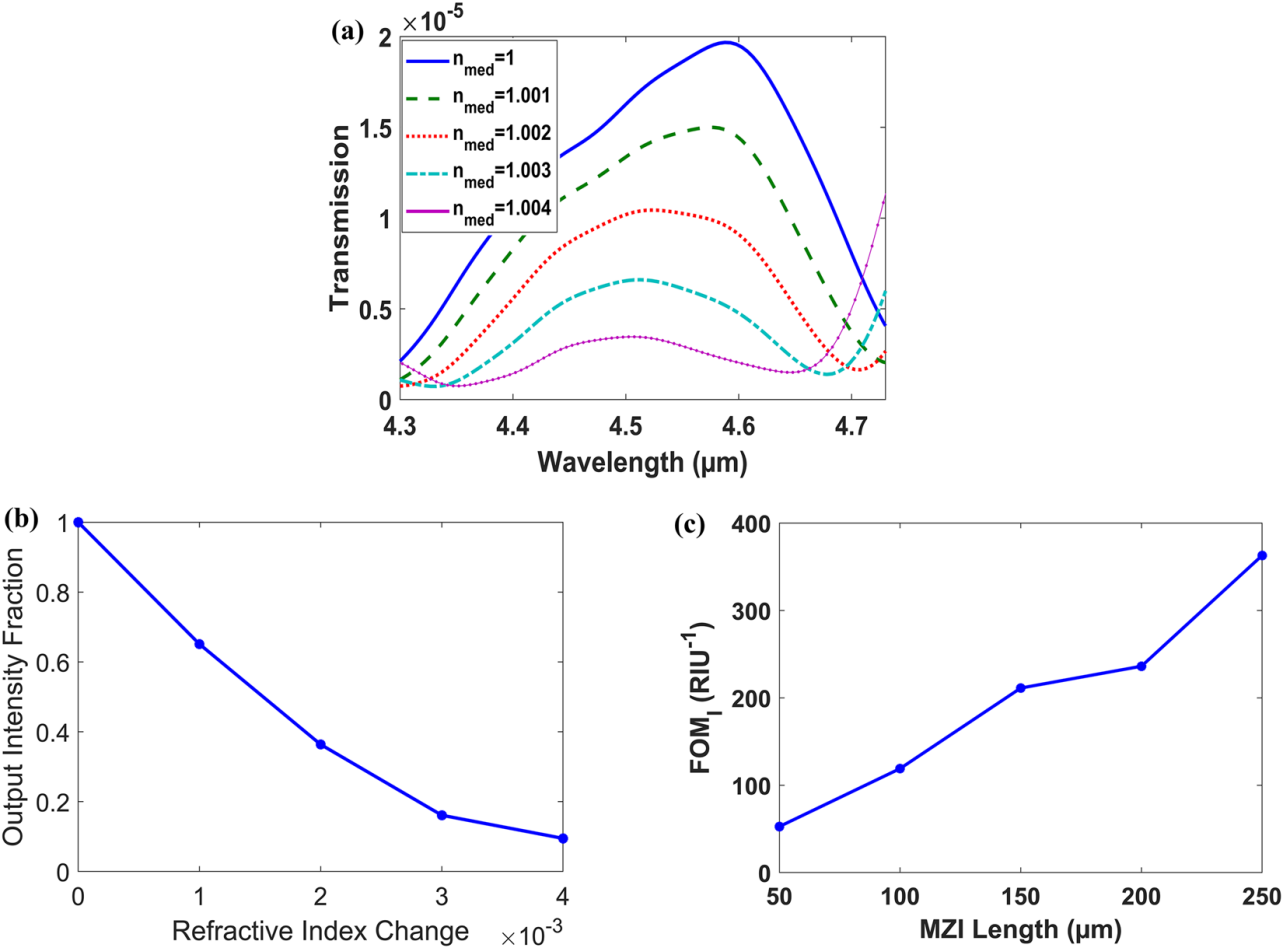
Vertical plasmonic MZI with D = 1000 nm, T = 2000 nm, h_HIL_ = 320 nm, w_1_ = 2100 nm, w_2_ = 1800 nm, P_gr_ = 918.4 nm and h_gr_ = 450 nm. (**a**) Transmission spectrum at different medium indices at L = 250 µm. (**b**) Output Intensity Fraction around λ = 4.6 µm versus air medium refractive index change (Δn_med_) at L = 250 µm. (**c**) Intensity interrogation FOM_I_ versus MZI length around λ = 4.6 µm.

Figure 9a shows the transmission spectrum at different medium indices, while Fig. 9b shows the output intensity change around λ = 4.6 µm versus the surrounding medium refractive index change, both at L = 250 µm. The FOM_I_ for different MZI lengths around λ = 4.6 µm is shown in Fig. 9c. This design shows much higher FOM_I_ than the previous design, in Fig. 8, 363 compared to 239 at L = 250 µm.

Note that, the fabrication of such structures is not challenging. Although the silver layer is thick around 2 µm the slots are also wide, minimum of 1.5 µm, hence the fabrication is simple as the aspect ratio is still small (almost one). The work in^38^ and^39^ shows that they easily patterned a 20 µm and 40 µm thick silver layers with even much higher aspect ratio (up to 5:1). In addition there are different etchant mentioned in^40^ which some of them are selective to silver, other selective to sapphire and other selective to silicon nitride. It is also important to note that, the effect of the fabrication tolerances is lower in our case since the dimensions and operating wavelength are large^41^.

## Conclusion

A free space coupled plasmonic MZI gas sensor operational in the MIR spectral region is designed and optimized. Using Si_3_N_4_ layer in the metal-insulator arm increases the sensitivity of the sensor to 10000 nm/RIU. Two different designs have been developed and proposed. The first design is optimized for maximum wavelength interrogation, FOM_λ_ = 133RIU^−1^, which also exhibits high intensity interrogation, FOM_I_ = 239RIU^−1^. While, the second design is optimized for maximum intensity interrogation, FOM_I_ = 363RIU^−1^, with low sensitivity to wavelength variations, both with L = 250 µm at λ = 4.6 µm. Intensity interrogation scheme have the advantage of low cost and compact size sensors. Using our sensor near the absorption fingerprints of the gases to be detected results in high performance sensors with low detection limits. Finally, the proposed sensors allow for mass-scale fabrication hence, low cost devices that are capable for real-time and high throughput sensing using multiplexed sensor arrays.
